# Prevalence, species composition, and associated risk factors of small ruminant gastrointestinal nematodes in South Omo zone, South-western Ethiopia

**DOI:** 10.5455/javar.2021.h550

**Published:** 2021-11-01

**Authors:** Tegegn Tesfaye

**Affiliations:** Southern Agricultural Research Institute, Jinka Agricultural Research Center, Livestock Research Directorate, Jinka, Ethiopia.

**Keywords:** Nematode infestation, endoparasites, nematodiosis, South Omo zone

## Abstract

**Objective:**

This study was conducted to generate data on the prevalence, species composition, and factors associated with small ruminant nematode infection in the South Omo zone, in South-western Ethiopia.

**Material and Methods:**

A cross-sectional study through fecal nematode parasite examination was conducted. Simple floatation test and coproculture, followed by Baermann technique, were used for nematode egg and third-stage larvae (L3) separation and identification. The McMaster method was used to calculate the egg per gram of feces (EPG).

**Results:**

From a total of 242 sheep and goats examined, 72.34% were infested with single or mixed nematode parasites. District, “Kebele”, species, body condition score, and age were significantly (*p *< 0.05) associated with nematode infestation. Simple logistic regression analysis indicated that nematode infestation in Bena-Tsemay district (78.33%) was significantly (*p *< 0.05) higher by a factor of 0.54 (OR 95% CI: 0.30–0.96) than Hamer district (66.39%). Among the species, caprine (79.43%) was significantly (*p* < 0.05) infested than ovine (62.37%) by a factor of 0.45 (OR 95% CI: 0.25–0.81). Moreover, infestation on poor (79.12%) and medium (70.96%) body conditioned animals was higher by a factor of 2.94 (OR 95% CI: 1.41–6.26) and 1.76 (OR 95% CI: 0.88–3.53) than on good body conditioned animals (63.79%). Likewise, infestation in age groups of 1–3 years (78.66%) and >3 years (84.40%) was significantly (*p *< 0.05) higher by a factor of 4.83 (OR 95% CI: 2.31–10.46) and 8.23 (OR 95% CI: 3.98–17.75) than younger age groups (41.37%), respectively. A moderate parasitic burden was observed on 52.90% of gastrointestinal nematodes (GIN)-infested animals with significantly (*p *< 0.05) higher EPG in females than males. Furthermore, mixed infestation of *Trichostrongylus axei* and *Eimeria* (6.19%), *Haemonchus contortus *and *Eimeria* (5.78%), and *Trichostrongylus vitrines *and *Eimeria* (5.78%) were dominantly identified. On the contrary, *T. axei* (15.70%), *Eimeria (*8.67%), *H. contortus (*7.43%), and *Trichostrongylus colubriformis *(7.02%) were dominant single infestations.

**Conclusion:**

The current study revealed the highest prevalence of GIN in the study area, which needs strategic control, needs to enhance community awareness toward GIN control and prevention, and to implement further investigation into anthelminthic efficacy to solve the problem.

## Introduction

Infections with gastrointestinal nematodes (GINs) are a significant impediment to livestock production in the tropics and elsewhere [1]. It significantly reduces smallholder farmers’ incomes through mortality, morbidity, and financial loss associated with treatment [2,3]. It has a greater impact in Sub-Saharan Africa due to the availability of a diverse range of agro-ecological factors suitable for diverse hosts and parasite species [4], inadequate nutrition of the host animal [5,6], and inadequate sanitation in rural areas [7,8].

Nematode parasites have varying degrees of pathogenicity [4]. The most economically significant endoparasitic diseases are gastrointestinal and respiratory nematodiosis, fascioliosis, and cestodiosis [1]. In general, these infections cause anorexia, decreased food intake, loss of blood and plasma proteins into the gastrointestinal tract, alteration of protein metabolism, enteritis, and diarrhea, all of which result in decreased body weight gains, wool growth, reproduction, and death due to secondary infections [4,9].

Despite their prominent economic contribution to poor rural communities of developing countries, the production and productivity of small ruminants are affected by different health constraints, among which gastrointestinal nematodiosis is the major one [10,11]. The gastrointestinal tract is the primary predilection site for harboring nematode parasites of small ruminants [12]. A wide range of either single or mixed nematode parasite infections is dominantly found in the abomasum, or small intestine, causing gastrointestinal nematodiasis. These include *Haemonchus*, *Cooperia*, *Ostertagia*, *Bunostomum*, *Trichostrongylus*, *Oesophagostomum*, and *Nematodirus* [12]. 

In the tropics, diseases caused by helminth parasites continue to be a significant impediment to small ruminant production [1]. In these latitudes, up to 95% of small ruminants are reported to be infected with helminths [13–15]. However, due to the chronic nature of the disease, the majority of animals infested with nematodes do not exhibit clinical signs. Due to the non-pathognomonic nature of nematode parasite infection, clinical diagnosis is extremely challenging [16,17], which has an effect on the recommended treatment for specific nematodiosis [16,18].

Small ruminants in Ethiopia are infested by different species of GIN, which cause both direct and indirect economic losses [10]. The host–parasite relationship and the prevailing agro-climatic conditions of the country play a vital role in the epidemiology of these nematodes. Several small ruminant nematodiosis prevalence studies have been conducted in various regions and agro-climatic zones of Ethiopia. These reports indicated a prevalence ranging from 50.4% to 84.1% [19]. The agro-ecological suitability of Ethiopia plays a leading role in the higher prevalence of GINs of small ruminants [10]. These reports also indicated age, sex, weather condition, husbandry, or management practices as some of many associated risk factors influencing the prevalence of small ruminant’s GIN in Ethiopia [10,20]. Despite numerous investigations conducted in the country, there is also scanty information on the prevalence and associated risk factors from different parts of the country, where large numbers of livestock populations are living. In such areas where small ruminants play a vital role in the community’s livelihood, the epidemiology of economically significant diseases such as nematodiasis is very important for control and prevention measures.

In South Omo zone, one of the leading pastoral areas in Ethiopia, small ruminants have paramount importance for the livelihood of the resident pastoral communities [21]. However, the production and productivity of small ruminants is limited due to poor genetic potential, different diseases, poor nutrition (in quantity and quality), and poor management and husbandry practices [11]. Among the diseases, GIN infections are suspected to be the most important causes of wastage and decreased productivity of the South Omo zone’s small ruminants, despite scant information. The conducive agro-ecology of the current study area presents suitable conditions for nematode parasites in the study area. Moreover, poor GIN management due to a lack of strategic deworming practice might play a vital role in the epidemiology of nematode parasites in the area. The lack of epidemiological data on small ruminant nematodiosis was the primary impediment to scheduling nematode control campaigns in the area. Therefore, the objectives of this study were to estimate the prevalence, species composition, and factors associated with the epidemiology of small ruminant nematodiosis in the area to generate baseline data for further control and prevention strategies.

## Materials and Methods

### Ethical approval

Ethical clearance for this study was obtained from the Southern Agricultural Research Institute (SARI) (SARI-07-008/2018). All ethical issues were considered during fecal sample collection from the study animals. For further compliance with ethical standards, mutual consent was made between the animal owners and investigators by briefing the study’s objective. All GIN-positive animals were dewormed after laboratory confirmation. 

### Description of the study areas

This study was conducted in Ethiopia’s South Omo zone, Southern Nations, Nationalities, and Peoples Region. This zone is delimited by international borders from the South by Kenya and from the Southwest by the Ilemi Triangle. Similarly, the zone shares a border with the Bench Maji zone from the West; Kefa zone from the Northwest; Gamo zone, Gofa zone, Konta, and Basketo special districts from the North; Derashe special district and Konso zone from the Northeast; and the Oromia region from the East. The South Omo zone has a total area coverage of 24,249 km^2^. Moreover, the Central Statistical Agency of Ethiopia survey data indicated the total cattle, sheep, and goat population of 1.75 million, 1.55 million, and 2.88 million, respectively, in this zone [22].

**Figure 1.**
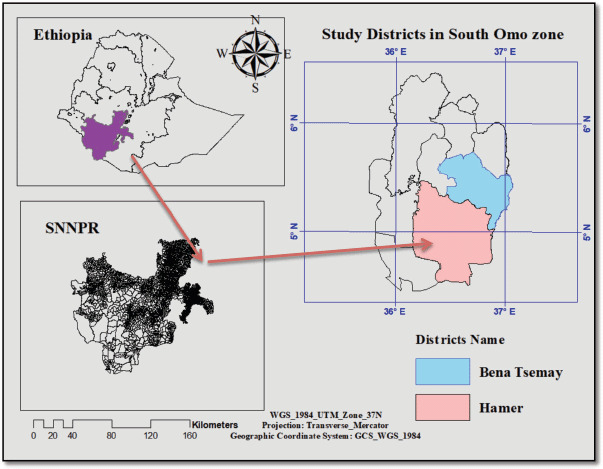
Map of the study zone (left bottom) and specific study districts (right).

Two districts (Bena-Tsemay and Hamer) were randomly selected from the South Omo zone (Fig. 1). The study Kebeles were Area Ambule and Besheda from Hamer district and Diziaman and Luka from Bena-Tsemay district. Bena-Tsemay district is 1 of the 10 districts in the South Omo zone with latitude ranges between 5° 00′ 31″ N and 5° 41′ 47″ N and longitudes range between 36° 12′ 13″ E and 37° 03′ 50″ E.

The altitudinal range of the Bena-Tsemay district varies between 500 and 1,800 m above sea level. Within this altitudinal range, the climate varies from warm to hot semi-arid. The mean annual rainfall of the Bena-Tsemay district is 1.400 mm, and the average daily temperature ranges from 15.6°C to 26.5°C. Whereas Hamer district is astronomically situated between 4° 0.50′–5°0.47′N and 36°.15′–36°0.90′E with an altitude that varies from 450 to 1,765 m above sea level. The mean annual temperature ranges between 29°C and 38°C, with an average yearly rainfall of 400 mm.

### Study animals and their management

Local sheep and goats (Woyto-Guji goat breed) were sampled for this parasitological study. The rangeland-based extensive management system is dominantly practiced by pastoralists and agro-pastoralists of the study area to manage their herds. The housing of small ruminants is barely practiced in pastoral areas of the South Omo zone, except for woody crashes devoid of roofs to avoid animals’ movement during nighttime. Shortages of quality and quantity of animal feed and water are the main problems of herd owners, especially during the dry season, which forces animals to travel long distances to search for feed and water. To tackle extreme water shortages, pastoralists and agro-pastoralists use community bore holes, locally known as “chirosh”, which are bored near large rivers after they dry out. Classifying and supplementing a group of animals (draft oxen, milking cows/goats, and calves) were rarely carried out in pastoral areas of the South Omo zone.

### Study design and methods of sampling

A cross-sectional study design was followed to establish the prevalence and associated risk factors of endoparasites in the study area. From the list of districts in the zone, two randomly selected districts (Bena-Tsemay and Hamer) were used for this study. From each district, two study villages (“Kebeles”) were randomly selected. Then, the simple random method was used to determine representative small ruminant owners from the villages listed on village administration. Herds kept by livestock owners were stratified based on species (goat and sheep), sex, age, and body condition score (BCS). Systematic sampling was followed to draw study animals from the selected herd (strata) by taking every animal chosen based on the calculated interval between first and second and then *n*th, depending on the selected herd. 

### Sample size 

There was no previous study on small ruminant endoparasites’ prevalence in the area. However, due to the seasonal mobility of pastoralists in search of water and pasture (since our study period coincided with the dry season), only 242 animals (101 ovine and 141 caprine) were randomly sampled from the study districts. This implies that a sample size of 384 is required with 50% expected prevalence at the desired accuracy level of 5% at the 95% confidence level [23].

### Laboratory analysis

A simple floatation test for GIN egg separation and identification was primarily applied to the method. Only a few nematode parasites (*Nematoirus* spp, *Trichuris* spp, *Strongyloides* spp) have characteristic eggs to differentiate them at the genus level. However, Strongyle-type nematodes (of the genus T*richrostrongyle* and *Strongyles*) are not easily distinguished by their eggs. Therefore, coproculture of composite (pooled) fecal samples was conducted to differentiate them at the genus level [17,24]. For the sake of precision, different sizes (5, 10, 15, …, etc.) of pooled samples were prepared from nematode positive cattle, depending on the number of positive parasite animals. The pooled fecal samples were finely broken up by using a stirring device after moistening them with water. Then, the prepared fecal samples were transferred to petri dishes and kept in an incubator having a temperature of 22°C –27°C and 85%–90% humidity for 7–10 days. Water was added regularly every 1–2 days to the fecal culture to avoid drying out of the culture during these days. Finally, the third-stage larvae (L3) and/or when present, L2 larvae were collected by using the Baermann technique, counted, and subjected to differentiation as per the key mentioned in a previous study [24]. The larvae’s (L3) key morphological features of the caudal and cranial extremities were examined for differentiation. Determination of the egg per gram of feces (EPG) and oocyst per gram of feces (OPG) was carried out using the McMaster technique [17]. The presence of oocytes and *Monezia* spp ova was also recorded. Strongyle infections were divided into three categories: low (200 EPG), moderate (201–700 EPG), and high (>700 EPG) [9].Similarly, coccidian burden was estimated by counting an oocyst, and its levels were arbitrarily divided into negative (zero), low (1–1,000 OPG), moderate (1,000–5,000 OPG), and high (>5,000 OPG).

### Data analysis

Data from fecal parasitological examinations were coded and entered into Microsoft Excel spreadsheets to create a database, which was then imported into SPSS version 20 for descriptive analysis.The prevalence was calculated by dividing the number of parasite-infested animals by the total number of animals examined. An independent sample test was implemented to establish whether or not there were significant differences in mean EPG among animal species, sex, and type of infestation. Simple logistic regression analysis via the R-software package was used to see the effect of each risk factor and their association with the prevalence of GINs. A 5% global significance level was used to carry out the tests. 

## Result

### GIN overall prevalence, type of infestation, and association with study areas 

Out of 242 small ruminants (101 ovine and 141 caprines) examined for endoparasites infestation, 175 caprine (79.43%) and ovine (62.37%) were found to be infested with single and/or mixed infestations, which results in an overall prevalence of 72.34% (Table 1). Of the total examined animals, 104 (42.97%) were infested with single helminth parasites, whereas the remaining 71 (29.33%) were co-infested (mixed infestation) with different types of endoparasites (Table 1).

According to the current study, the prevalence of nematode parasites in the study area was significantly associated (*p* < 0.05) with study districts and Kebeles (Table 2). The simple logistic regression analysis indicated that prevalence in the Hamer district (66.39%) was lower than Bena-Tsemay district (78.33%) by a factor of 0.54 (OR 95% CI = 0.30–0.96). Similarly, the GIN prevalence in Besheda, Diziaman, and Luka Kebele was higher by factors of 6.04, 1.84, and 14.93, respectively, than its prevalence in Area Ambule Kebele (Table 2).

**Table 1.** Overall prevalence and type of infestations.

Description	Total examined animals	No. of +ve animals	% of +ve	*p*-value
Single infestation	242	104	42.97	0.99
Mixed infestation	242	71	29.33	
Overall prevalence	242	175	72.34	

### Association of small ruminant GIN infestation with host-related risk factors

There was a significant (*p* < 0.05) variation in GIN prevalence among the species. It was higher by a factor of 0.45 (OR 95% CI = 0.25–0.81) on caprine (79.43%) than ovine (62.37%). Although there was insignificant variation in prevalence among the sexes, a slightly higher prevalence was recorded in females (73.29%) than in males (69.69%) (Table 3). Moreover, animals with poor body condition (79.12%) were more and significantly (*p* < 0.05) infested by GINs than animals with medium (70.96%) and good body condition (63.79%). In poor body conditioned animals, the prevalence was higher by a factor of 2.94 (OR 95% CI = 1.41–6.26) compared to animals with good body condition (Table 3).

Furthermore, GIN prevalence among age groups was significantly (*p* < 0.05) varied. It was higher in age groups of >3 years (84.40%) and 1–3 years (78.66%) as compared to young age groups (<1-year-old) (Table 3). Simple logistic regression analysis indicated that endoparasite prevalence was higher by factors of 4.83 (OR 95% CI = 2.31–10.46) and 8.23 (OR 95% CI = 3.98–17.75) respectively, on 1–3-year-old and >3-year-old animals as compared to the <1-year-old group.

### Species composition and parasitic burden of dominant GIN infestation in the study area

*Trichostrongylus axei *(*T. axei*) and* Eimeria *(6.19%),* Haemonchus contortus* and *Eimeria* (5.78%), *T. vitrinus* and *Eimeria* (5.78%), and *Trichostrongylus colubriformis* and *Eimeria* (2.48%) were dominantly identified. *Trichostrongylus axei* was dominant as a single infestation in the area, followed by *Eimeria*, *H. contortus*, and *T. colubriformis* (Table 4).

As revealed by this study, 8.38%, 52.90%, and 38.70% of GIN positive small ruminants harbor light, moderate, and high levels of parasite burden, respectively (Table 5). However, the oocyst count indicated that coccidian parasites lightly infested all study animals.

**Table 2.** Endoparasites association with study districts and kebeles.

Risk factors	Category of risk factors	No. of examined animals	% of +ve	Simple logistic regression		
				OR	OR 95% CI	*p*-value
District	Hamer	122	66.39	0.54	0.30–0.96	0.039*
	Bena-Tsemay	120	78.33			
Kebele	Area Ambule	62	48.38			
	Besheda	60	85.00	6.04	2.62–15.06	0.000*
	Diziaman	60	63.33	1.84	0.89–3.83	0.097
	Luka	60	93.33	14.93	5.32–53.73	0.000*

OR = Odds ratio; CI = Confidence interval.

* = Statistically significant.

**Table 3.** Association of endoparasites prevalence with different host-related risk factors.

Risk factors	Category of risk factors	No. of examined animals	% of +ve	Simple logistic regression		
				OR	OR 95% CI	*p*-value
Species	Ovine	101	62.37	0.45	0.25–0.81	0.007*
	Caprine	141	79.43			
Sex	Male	66	69.69	0.75	0.41–1.42	0.38
	Female	176	73.29			
BCS	Poor	91	79.12	2.94	1.41–6.26	0.004*
	Medium	93	70.96	1.76	0.88–3.53	0.105
	Good	58	63.79			
Age	<1 years	58	41.37			
	1–3 years	75	78.66	4.83	2.31–10.46	0.000*
	>3 years	109	84.40	8.23	3.98–17.75	0.000*

OR = Odds ratio; CI = Confidence interval.

* = Statistically significant.

Mean egg count (EPG) of parasite-positive small ruminants showed a statistically significant (*p* < 0.05) association with sex, which was higher in female animals than males (Table 6). However, the species of study animals and type of infestation were insignificantly associated with mean EPG (Table 6).

## Discussion

The results of this study indicated an overall small ruminant GIN prevalence of 72.34% in the study area. This indicated the presence of conducive environmental conditions for the epidemiology of small ruminant GIN parasites in the area. This result is in line (nearly similar) with previous reports from Northern Ethiopia [25] and Western Oromia [19], who reported a GIN prevalence of 70.6% and 69.6%, respectively. The current meta-analysis report of gastrointestinal nematode infection (75.8%) in small ruminants by Asmare et al. [10] is also in agreement with the current prevalence report. 

However, the current finding was higher than the previous prevalence of 43.2%, 63.4%, 51.4%, 53.9%, and 40.9%, which were reported, respectively, by Dembia [26], Debre Zeit Elfora [27], Gamo Gofa [28], Tullo district [29], and Wukro [30]. Higher GIN prevalence in the current study area compared to previous studies might be due to poor health management (deworming practice), high stocking density, co-grazing of ruminants, and differences in agro-ecology. Besides, the poor animal nutritional practice of the current study area may favor further infection burden because lack of optimized nutrition might decrease the ability of animals to cope with the adverse effects of worm infestation [5,6,31]. 

Moreover, our finding was lower than previous reports of 87.8%, 77.8%, 87.5%, 84.4%, 92.9%, 77.4%, and 79.6%, respectively, from Afar [32], Bale [33], Guto Gida district [34], Hawassa [35], Ogaden region [36], Wolaita Soddo [37], and six districts of West Oromia [19]. This might be due to either a difference in diagnostic techniques used by the authors or a difference in the number of animals examined for the study.

Significant prevalence differences among the study districts and Kebeles might be due to variation in deworming practices of herd owners in each study site. It might also be due to differences in environmental conduciveness among the study sites. According to our findings, caprines were highly and significantly more infested (0.45 times) than ovines. This is in agreement with findings from the eastern part of Ethiopia [38], Dembia district [26], and Bale zone [33]. Higher infestation of caprine might be attributed to their vigilant nature at grazing sites as compared to sheep, in which they make up the front line of the herd, which allows them to get more parasite infestation. However, reports by Mohammed et al. [28] from the Gamo Gofa zone and Tesfaheywet and Murga [39] from Hawassa were inconsistent with current findings that declared a significantly higher prevalence of ovine than caprine. Moreover, Kenea et al. [40] confirmed insignificant infestations among the species from the Kaffa and Bench Maji zones. 

**Table 4.** Major mixed and single type infestations.

Types of mixed infestations	No. of examined animals	No. Positive animals	%
*Trichostrongylus axei *and* Eimeria*	242	15	6.19
*Haemonchus contortus *and* Eimeria*	242	14	5.78
*Trichostrongylus vitrinus *and* Eimeria*	242	14	5.78
*Trichostrongylus colubriformis *and* Eimeria*	242	6	2.48
*Trichostrongylus axei*	242	38	15.70
*Eimeria*	242	21	8.67
*Haemonchus contortus*	242	18	7.43
*Trichostrongylus colubriformis*	242	17	7.02

**Table 6.** Mean of EPG of parasites in positive fecal samples per animal species, sex, and type of infestation.

Risk factors	Category of risk factors	Mean EPG	± SE	*p*-value	95% CI	
					LB	UB
Species	Caprine	668.63	36.47			
	Ovine	746.30	53.59	0.22	−203.14	47.80
Sex	Male	527.50	41.79			
	Female	753.45	36.63	0.001*	−358.52	−93.38
Type of infestation	Single	673.84	36.47			
	Mixed	722.14	50.532	0.43	−168.66	72.04

SE = Standard error; CI = Confidence interval; LB = Lower bound; UB = Upper bound.

* = Statistically significant.

In this study, insignificant prevalence among sex might be because of the equal chance of infestation in both sexes as they share the same grazing site. Consistent findings were revealed from Northern Ethiopia [25], Gamo Gofa [28], and Dembia [26]. However, doe and ewe are more susceptible to and infected with nematode parasites than males during physiological conditions, such as pregnancy, parturition, and lactation [41–43]. 

According to the current study, nematode infestation was significantly associated with BCSs. As indicated, animals with poor body conditions were more severely infested than animals with medium and good body conditions. This is in agreement with previous scholars’ conclusions from Burie district [44], Kaffa and Bench Maji zones [40], Tullu district [29], and Bedelle [45]. However, the contradictory conclusion of Dabasa et al. [33] from the Bale zone stated that well-conditioned animals were more prone to GIN infestation than poor and medium-conditioned ones. Previous studies [31,46] have stated that higher infestations in poor body condition animals might be attributed to compromised immunity due to other diseases and deprived nutritional status, which could expose them exceedingly compared to animals with good and medium body condition.

The current study found that adult animals had significantly more GIN infestation than young animals.This might be because young kids concentrate on suckling their ewes and does rather than grazing and/or browsing, which could decrease their exposure to infestation. Similar findings were declared in different parts of Ethiopia [33,40], Malaysia [47], and Lesotho [48]. However, other researchers from Ethiopia [49] and abroad [50,51] contrasted with this finding and discovered a higher prevalence of young animals than adults. Most of these contrasting scholars stated the effect of acquired immunity of adult animals due to repeated exposure to infestation, which helps in expelling the new infestation with resultant low prevalence. 

The result of coproculture has shown the dominance of Strongyle-type nematodes (*T. axei, H. contortus, *and* T. colubriformis*) infestation in the study area. The previous study of Yimer and Birhan [25] agreed with our finding. They identified *Haemonchus* (9.9%) and *Trichostrongylus *(6.5%) as the most predominant genera of Strongyle-type of nematodes of small ruminant nematodiasis causing single infection in their study area. Our current finding was also agreed with the previous reports of Bersissa and Ajebu [35] in Hawassa, Mbuh et al. [14] in Cameroon, and Tariq et al. [52] in the Kashmir valley, who all reported the predominance of Strongyle-type nematodes (especially of the genus *Haemonchus*) in small ruminants.

Significantly higher egg shedding by female animals than males was observed in our current investigation. This is in agreement with a previous report from Ethiopia [53,54] and India [55]. Higher EPG by female animals might be due to immunosuppression associated with pregnancy and periparturient periods, resulting in heavy nematode burdens, as stated by Kahn et al. [41]. Getachew et al. [56], Mushonga et al. [57], and Poddar et al. [58] from Ethiopia, Rwanda, and Bangladesh, respectively, discovered statistically insignificant EPG among the sexes. 

## Conclusion

This study concluded that gastrointestinal nematodiasis constitutes a serious handicap to small ruminant production in the current study location. The predominant etiological agents were single and mixed infestations of Strongyle-type of genus *Trichostrongylus* and *Haemonchus,* and to a lesser extent, of other genera, including *Eimeria* and *Monezia*. Among the studied risk factors, study districts, study “Kebeles” (villages), species, BCS, and host’s age were significantly associated with GIN infestation of small ruminants. Agro-ecological suitability, poor deworming practice, co-grazing and high stocking of ruminants, poor nutritional management, and concurrent diseases were suspected as encouraging factors for the high prevalence of nematode infestations in small ruminants. Therefore, strategic intervention practices through periodic deworming should be designed by considering all conducive factors for nematodiasis. Moreover, community awareness of nematode infestation treatment, prevention, and control are very important to curb the problem. Furthermore, studies should be conducted to explain seasonal variation (if any) in the prevalence and effectiveness of commonly used anthelmintics in the area.

## List of Abbreviations

BCS: Body condition score, EPG: Egg per gram of feces, GINs: Gastrointestinal nematodes, OPG: Oocyst per gram of feces, SARI: Southern Agricultural Research Institute.
